# 557. Utilizing the Burn Care Quality Platform to Examine Diabetes and Infectious Outcomes After Burn Injury

**DOI:** 10.1093/jbcr/irag033.297

**Published:** 2026-04-06

**Authors:** Jacob Furcolo, Arthur Kom Sipowa, Tuan D Le, Melissa M McLawhorn, Bonnie C Carney, Lauren T Moffatt, Jeffrey W Shupp, Shawn Tejiram

**Affiliations:** The Burn Center at MedStar Washington Hospital Center, Washington, DC; The Burn Center at MedStar Washington Hospital Center, Washington, DC; The Burn Center at MedStar Washington Hospital Center, Grand Prairie, TX; MedStar Health, Washington, DC; The Burn Center at MedStar Washington Hospital Center, Washington, DC; The Burn Center at MedStar Washington Hospital Center, Washington, DC; MedStar Washington Hospital Center, Washington, DC; MedStar Washington Hospital Center, Washington, DC

## Abstract

**Introduction:**

Diabetes mellitus (DM) in burn patients is associated with delayed wound healing, immune dysregulation, and higher perioperative risk. This increased physiologic distress may correlate with increased infection risk. Prior literature examining the association of DM with infection risk in burn patients often relies on smaller cohorts or narrow endpoints. Using a large, multicenter database like the Burn Care Quality Platform (BCQP) may solicit heretofore unknown risks or associations with infection in burn patients with DM. This work aimed to evaluate rates of infection between burn patients with and without DM and associated outcomes. A secondary analysis of blood glucose control following admission was also examined as a potentially modifiable factor.

**Methods:**

The BCQP platform was used to retrospectively analyze burn patient data from 2016 to 2018. Patients were stratified into diabetic (DM) and nondiabetic (NDM) groups. Primary outcomes examined included ventilator-associated pneumonia (VAP), central line-associated bloodstream infection (CLABSI), and acute respiratory distress syndrome (ARDS). Secondary outcomes examined were in-hospital mortality, hospital length of stay (LOS), ICU LOS, and ventilator days. Continuous variables were reported as median (interquartile range) and compared with the Mann–Whitney U test. Categorical variables were compared with chi-square or Fisher’s exact tests. Multivariable logistic regression was specified to evaluate associations between DM and outcomes. Analyses used SAS 9.4 and Prism 10; significance level set at α = 0.05.

**Results:**

Of 3797 patients, 856 (22.5%) had DM. Patients with DM were mostly male (70.4%) and White (61.5%) with a median age of 50 years (IQR, 34-62). Median total body surface area (TBSA) burned was 4.2% in NDM vs. 3.8% in DM patients (*p*=.002). DM patients exhibited a higher proportion of hyperglycemia at both 24 hours (28.3 vs 4.5%) and 48 hours (25.5 vs 2.7%) following admission (*p*<.0001). Rates of VAP (2.5% vs 2%, *p*=.6673), CLABSI (1.5% vs 1.4%, *p*=.7277), and ARDS (2.1% vs 1.%, *p*=.2177) demonstrated no differences among cohorts. However, DM had longer LOS (8 vs 5 days, *p*<.0001) and higher in-hospital mortality (5.7% vs 4.1%, *p*=.045).

**Conclusions:**

Patients with DM did not have significantly higher rates of ARDS, VAP, or CLABSI. However, patients with DM experienced significantly longer hospitalization and higher in-hospital mortality. Further work will be necessary to better elucidate these findings.

**Applicability of Research to Practice:**

This novel multicenter analysis supports early identification of burn patients and protocol-driven management that emphasizes glucose monitoring, optimization, and proactive discharge planning to offset longer LOS.

**Funding for the study:**

N/A.

